# The Food and the Teeth

**Published:** 1865-11

**Authors:** James Paul

**Affiliations:** Trenton, N.J.


### Selections.

### THE FOOD AND THE TEETH.—Observations on the Inorganic Constituents of the Food of Children, as connected with the Decay of the Teeth, and the Physical Constitution of Women in America.

By the late JAMES PAUL, M.D., of Trenton, N.J.

[*Concluded from page* 634.]

You will observe that the albuminous or nutrient differs from the saccharine and oleaginous, in containing nitrogen, and sulphur and phosphorus, with carbon, hydrogen, and oxygen, while the latter contains only carbon, hydrogen, and oxygen—nitrogen being required in those compounds which give strength and formation to the frame.

Now, the albuminous, or nutritive, being that portion which affords nourishment to the body, contains those constituents required in the first place for the formation and giving strength to the different portions of the body, and, when fully developed, of repairing the general waste continually going on in the system, whether from the usual wear and tear, fractured bones, or the ravages of disease. And the saccharine and oleaginous—the calorifacient or heat making—to keep up a continual supply of fuel, as it were, that the body may be kept of a regular and proper temperature; for you are, no doubt, aware that there is a continued supply of carbon, or, in more simple language, of charcoal, required to keep up the natural temperature of the body; and what is not required for immediate use is stored away in the form of fat, to be called into action as occasion requires.

We have seen in the analysis of milk, that the fluid contains butter, cheese, and sugar; consequently, we can understand how an infant can thrive so well upon it—the cheese or caseine* of the milk containing the nitrogenized or nutrient principle, which, together with the earths and salts contained in the milk, goes to form the bones, muscles, and the different tissues of the body—the sugar, which we have seen by the analysis, contains a large quantity of carbon in its composition, going to keep up the temperature of the infant, while the butter, in the nature of fat, is stored away in a healthy infant, filling up every vacant interstice, causing a roundness and plumpness, the pride and joy of the happy parent.

Now let us mark the difference of the babe that has been denied a milk diet, and is doomed, by ignorance, to be fed on starch and sugar. You will recollect that these two substances were composed of carbon, hydrogen, and oxygen only. By a process of digestion which I need not here enter into, such food is converted into sugar, the carbon of which becomes the fuel by which the temperature of the body is kept up—there being no principle in the food to give albumen, there is nothing taken into the stomach upon which the gastric fluid can expend its solvent powers; the infant is, therefore, much troubled with acid eructations, and the stomach becomes weak and irritable. The want of the nutritive constituent of the food, and the earths and salts, etc., necessary and essential for the formation of the bones and teeth, show a lamentable deficiency in the child's development, and there being no fatty matter to be laid up, the body is emaciated, the countenance is ghastly, the flesh and integuments hang soft and flabby over the bones, no absolute disease can be detected, the child is ravenous and hungry, and the unfortunate babe descends to the tomb, a spectre and an object of the most pitiful description. This is no fancy sketch, but one too often met with in the ordinary walks of professional life. And why is it so? Simply because the composition of the human frame, the component parts of our food requisite to produce that frame, and the process of digestion and nutrition are so little understood.

We now advance from infancy to childhood—and this is a

| * | Analysis of Caseine from fresh milk. | Albuminous substances found in whey, after coagulation with an acid. |
|---|---|---|
| Carbon | 54.825 | 54.96 |
| Hydrogen | 7.153 | 7.15 |
| Nitrogen | 15.628 | 15.89 |
| Oxygen } | 22.394 | 21.73 |
| Sulphur } | | 0.36 |

period when the greatest attention is required in supplying nutriment to aid nature in the great work of developing the body. The child is now deprived of the maternal secretion, and dependent on food prepared for its use by the hand of man—perhaps living in a city, and deprived of pure wholesome milk from the cow. And we know there is a vast disproportion in the quality of milk when the cow is country-fed on the natural productions of the farm, and when city-fed on slops and grain, the refuse of the brewery.

It is at this age that the great proportion of bony substance is deposited; those of the extremities are lengthened, become more compact and stronger, and the substance of the teeth is deposited in the cells of gelatinous tissue. How necessary is it, then, that this subject should receive the utmost attention of parents. It has hitherto been too much the custom to leave all this, as belonging entirely to nature—as a thing we had nothing to do with. We have been too much in the habit of considering that nature furnished her own materials, and man had nothing to do with her operation. The potter cannot fashion the bowl without the clay, neither can bone be formed without earth. No, my friends, nature must be supplied with the material, which, although offered in the most incongruous forms, she has the power of decomposing, selecting from, and supplying for the various purposes required; one portion, as we have already stated, to act as fuel in keeping up the temperature; another portion she selects to add to the flesh, the muscle, skin, and different tissues; and the earths which are held in solution, she carries away by vessels adapted for that purpose, and deposits them, atom by atom, until they are so compressed, so strongly compacted together, as to become what we call *solid bone;* and all this so wonderfully wrought, that, as we have seen, small tubes are left in the hard, stony formations both of the bones and of the teeth, that nourishment may be supplied them, holding in solution the material of which they are composed, that natural waste and decay may be replaced, and injuries repaired.

It is to this nutrition, and of the earthy matter of which the bones and teeth are composed, a deficiency which is attended with results so deplorable, that I particularly wish to call your attention.

To what can we attribute the calamity which too often befalls the young? I allude to distorted spines, where the bones composing the spine, instead of forming a column, allowing the body to be erect and dignified, are zigzag in their course, causing one shoulder to bulge out, and the opposite side to bend or double upon itself. This deformity has been long understood to arise from a deficiency of *lime* in the composition of the bones of the vertebræ, allowing them to fall, press upon, and injure each other, destroying the beauty of the fabric, and the health and comfort of the individual.

Now let us take a glance at the inhabitants of two countries, natives of which are no strangers on this continent. I take them as examples, because the food of the *common people* of those countries, is well known to be of the most common kind. I allude to the natives of Scotland and Ireland—the principal food of one being *oatmeal*, and of the other, potatoes. We have heard a great deal of the famishing poor of those countries, and particularly of the latter—of the misery and wretchedness seen in every hovel; and there cannot be a doubt that a famine walked through the land, when the blight and rot despoiled them of their potato crop, on which, for so long a period, they depended as the great article of food. Now, allowing all this—allowing in the *best seasons*, the chief article of subsistence has been potatoes for breakfast, dinner, and supper; glad indeed many of them to get a little animal food once a week to dinner or even far more seldom—I now ask, what number, in the thousands of emigrants from that country who yearly arrive at our ports, are there that show a constitution weak, fragile, and wanting in physical strength? Many, no doubt, arrive, worn down by disease and suffering, and in the last stage of debility; but let them recover from that state, and the robust frame and healthy constitution will be again developed; and the bones are strong, the teeth undecayed, and the muscular energy only wanting opportunity to display itself;—in fact, when we wish to denote strength in women, we use the familiar phrase, "strong as an Irish women;" and all this from being reared on *potatoes*. But then if we examine the analysis of the potatoes, we shall find contained in 100 parts of dry potatoes,—

| | |
|---|---|
| Carbon | 41.1 |
| Hydrogen | 5.8 |
| Nitrogen, Oxygen, | 45.1 |
| Ashes | 5.0 |

Here we see that potatoes not only contain the nutrient but the earthy constituents.*

* According to a memorial presented to the French minister, on the proportions of nutriment of the means of living, by Dr. Glaser, we find potatoes taking no mean rank.

But we have a stronger and more healthy race yet, from Scotland and the north of Ireland, who are generally descendants of the Scotch, and continue, in a great measure, the same means in rearing the young. Now, *a* principal, I will not say *the* principal food of the youth of Scotland, high and low, rich and poor, except in the larger cities, amongst those who class themselves as more refined and more civilized, but who number few in proportion, consists, for breakfast, at least, of oatmeal—that is, porridge and milk; and milk, potatoes, and wheaten, oaten, and peas bread, or *bannocks*, at other times of the day. Animal food amongst the poor is a rarity; a meat dinner on Sunday *only*, being common. Even, among the youth of the better class, butcher's meat, or animal food, is by no means a principal article of subsistence. And I would particularly remark that *Scotch oatmeal* (the oatmeal generally used throughout Scotland) is coarse, and contains much of the bran which invests the oat—containing as it does, a large proportion of the earthy constituents required for the production of bone. Analysis of 100 parts of dried oats gives:—

| | |
|---|---|
| Carbon | 50.7 |
| Hydrogen | 6.4 |
| Oxygen | 36.7 |
| Nitrogen | 2.2 |
| Ashes | 4.6 |

I may here casually remark, that the advantage to be derived from this wholesome food has not escaped the observation of her majesty, Queen Victoria, who appears in the multiplicity of her public duties, not to lose sight of the equally sacred duties of a mother—and we hear of her son, the heir to the crown of Great Britain, being as fond of his oatmeal porridge as the meanest peasant child in Scotland.

I rather doubt, if parents generally have given to this subject the attention to which it is entitled. I trust, however, that those who have followed me thus far, may be impressed with its importance. We cannot shut our eyes to the complaint which so

NUTRITIVE ELEMENTS.

| | | | | |
|---|---|---|---|---|
| 100 lbs. | Wheat Bread | contains | 30 lbs. | |
| " | Flesh | " | 21 lbs. | |
| " | Fresh beans | " | 80 lbs. | casein and starch. |
| " | Peas | " | 83 lbs. | |
| " | Lentils | " | 94 lbs. | |
| " | Potatoes | " | 25 lbs. | albumen, starch, and sugar. |
| " | Carrots | " | 14 lbs. | albumen with sugar. |
| " | Beets | " | 8 lbs. | |

generally prevails, of decayed teeth—and a moment's reflection will call to mind the number of the young and beautiful who are prematurely hurried to the tomb, ere yet the bud has expanded into the full developed flower. Nay, comparing the two countries, the statistics of life and death communicate to us also the important fact, that while the greatest mortality shows itself in England in infancy and childhood, on this side the Atlantic, it is found at a more mature age.

Neither has the tendency of the physical organization of women on this continent to degenerate, escaped the observation of one of our greatest medical philosophers in this country.* who regards this retrogression as a national calamity, and impresses upon his students the importance of the subject, and the propriety of their attention in attempting to arrest it; and he particularly specifies the great object to be gained in the use of bran-bread, made from unbolted flour. On this head, I shall have more to say hereafter.

With these observations, let us now direct our attention to what can be offered in remedy of this evil.

We have already stated, that in no country in the world are children more beautiful or more lovely—healthy in complexion, quick, smart, and intelligent—active, sprightly, and playful in their disposition. Now, in the period from infancy until the child becomes mature—let us, at all events, say until thirteen or fourteen years, and even to a more advanced age—there is a continued growth—a continual deposition of organic and inorganic or earthy particles, which are required for the formation of the bone, teeth, flesh, and every part of the human body. I have shown you that the essential ingredients for these several formations are all found in the milk of the mother; consequently, as long as the infant is deriving nourishment from the mother, she ought to partake of good, wholesome, nourishing food—that the blood, deriving these principles from the food, may be able to supply them in turn to the milk from which it is secreted. So long then, as the child is thus nourished, so long is it safe, and the rudiments or foundation of a robust frame is laid. And if we are to expect, in future life, the stalwart frame of man, or the enduring, firmly-knit, compact, and healthy physical constitution in woman, the organic and inorganic or earthy compounds of which that frame is composed must not be denied—Nature must be supplied, or Nature will fail.

It is not for me to dictate to any parent what shall be the food of his child—it is enough that I point out for his informa-

* Dr. Jackson, of Philadelphia.

tion, what may be required to give, what in common language is called "bone and sinew," to their offspring. It is necessary then that the food of children should contain:—

1st. Aliment, having the *calorifacient* or heat-sustaining principle. And this is contained in quite sufficient quantity in the usual food—in milk, wheaten bread, potatoes, arrow-root, Indian corn, (as mush hominy, or corn-bread,) in most vegetable matter, and in sugar.

2d. Aliment containing the *nutrient* principle. And this is contained in animal food—the lean of beast, bird, and fish—in milk, eggs, wheat, rye, potatoes, beans, etc., etc.

And, 3d. Aliment containing the inorganic or earthy constituents—in which depends strength of frame, and from which are formed the bones and teeth of the individual. And these are contained in milk, eggs, animal food, and particularly in wheat, rye, oats, potatoes, etc.*

Of the inorganic constituents contained in wheat, (and the same may be said of the other *cereal* grains,) I have alluded to the benefit to be derived from using bread made of unbolted flour. On this subject, allow me to refer to the difference of flour having much of the bran remaining, and superfine flour, or that in general use throughout this country, and on which Prof. Johnston has made the following curious but practical observations. Examining wheat and flour, as to the amount of

* On this subject, I extract the following from Carpenter's Physiology, page 488:—"These substances are contained, more or less abundantly, in most articles generally used as food; and where they are deficient, the animal suffers in consequence, if they are not supplied in any other way. Thus, common *salt* exists, in no inconsiderable quantity, in the flesh and fluids of animals, in milk, and in eggs; it is not so abundant, however, in plants; and the deficiency is usually supplied to herbivorous animals by some other means. *Phosphorus* exists also in the yolk and white of the egg, and in milk—and it abounds, not only in many animal substances used as food, but also (in the state of phosphate of lime or bone earth) in the seeds of many plants, especially the *grasses*. In smaller quantities, it is found in the ashes of almost every plant. *Sulphur* is derived alike from vegetable and animal substances. It exists in flesh, eggs, and milk: also in the azotized compounds of plants; and (in the form of sulphate of lime) in most of the river and spring water that we drink. *Iron* is found in the yolk of egg, and in milk, as well as in animal flesh; it also exists is small quantities in most vegetable substances used as food by man—such as potatoes, cabbage, peas, cucumbers, mustard, etc. *Lime* is one of the most universally diffused of all mineral bodies; for there are few animal of vegetable substances in which it does not exist. It is most commonly taken in, among the higher animals, combined with phosphoric acid; in this state it exists largely in the seeds of most grasses, and especially in wheat flour. If it were not for their deficiency of *lime*, some of the leguminous seeds (peas) would be more nutritious than wheaten flour; the proportion of azotized matter they contain being greater. A considerable quantity of lime exists, in the state of carbonate and sulphate, in all hard water."

the nutrient or muscular matter, the fat-forming principle, and the bone and saline material, contained in grain in different States, he found that

| | Muscular Fat. | Fat Prin. | Bone & Sal. |
|---|---|---|---|
| In 1000 lbs. of whole grain | 156 lbs. | 25 lbs. | 170 lbs. |
| " fine flour | 130 lbs. | 20 lbs. | 60 lbs. |
| " bran | ------ | 60 lbs. | 700 lbs. |

Taking the three substances together, according to Professor Johnston, of 1000 lbs., the three substances contain, of the ingredients mentioned,—

| | Whole Grain. | Fine Flour. |
|---|---|---|
| Of muscular matter | 156 lbs. | 130 lbs. |
| Of bone material | 170 lbs. | 60 lbs. |
| Of fat | 28 lbs. | 20 lbs. |
| | 354 lbs. | 210 lbs. |

Accordingly, the whole grain is one-half more nutritious than fine flour.* It also shows the very great proportion of *bone material*,—that is, *earthy constituents*,—contained in the bran: no less than 700, out of 1000 parts, or a *little more than two-thirds* of the whole. Now, by reference to the same work, we find, in a communication from a Mr. Bentz, the difference in weight of a barrel of flour, without the bran, and when only the outer coating of the wheat is taken off. He says, "The weight of the bran or outer coating would, therefore, in the common superfine flour, constitute the *offal*, weighing only 5¼ lbs. to the barrel of flour, whilst the ordinary weight of offal is from 65 to 70 lbs. to each barrel of flour; showing a gain of from 59¾ to 65 lbs. of wheat in every barrel of flour." Now, if we estimate the earthy constituents to be two-thirds of the offal or bran, we must consider that there is an actual loss of these important constitutents, which might be reserved, in every barrel of flour, of 40 lbs.

*Again*, if we estimate, (according to the average of the consumption of flour to the amount of population, as one barrel to each individual,) that every child shall consume annually only half a barrel of flour, then we find, that by the use of the superfine flour, as commonly used in families, the child is deprived yearly of 20 lbs. of those earthy substances which are required to form the bones and the teeth. When we speak of a child consuming half a barrel of flour annually, it appears a large quantity; but when we reduce the same to a daily allowance,

* Patent Office Report, 1847, p. 116.

we find that it is little more than 4 oz. or 4½ oz.; and every parent must know that this would be a very small amount to limit children. Yet we see how large a quantity of the bony material would be added, if unbolted flour was used instead of the present superfine flour. I may here add, that the oatmeal used in Scotland, already referred to, contains the bran or inorganic constituents, while the oatmeal used in England is deprived of it. Now this is a great loss of the most valuable con-constituents in only one of the principal articles of the food of children; and if we allude to another article, which is largely used on this continent,—I mean Indian corn,—(and I may also add the fat of meat, both of which, children, if allowed, will partake of very freely,) we shall find that both of these abound more in the calorifacient, or heat-sustaining principle, and for the deposition of fat, than the nutrient; and that they are quite deficient of the earthy material, *of lime*—that material on which so mnch depends the proper structure of the teeth. Analysis of Indian corn shows the following composition—as taken from Mr. Salisbury's prize essay—read at the New York Agricultural Society, for 1849:—

Whole kernel.

| | |
|---|---|
| Starch | 50.64 |
| Sugar and Extractive | 7.46 |
| Sugar | 1.50 |
| Fibre | 6.28 |
| Matter separated from Fibre | 0.05 |
| Albumen | 8.64 |
| Caseine | 1.70 |
| Gluten | 4.56 |
| Oil | 4.00 |
| Dextrine or Gum | 4.84 |
| Water | 10.22 |
| | 99.89 |

Ash of the kernel constituting about two per cent.

| | |
|---|---|
| Carbonic acid | a trace. |
| Silicic " | 1.450 |
| Sulphuric " | 0.206 |
| Phosphoric acid | 50.955 |
| Phosphate of Iron | 4.355 |
| Lime | 0.150 |
| Magnesia | 16.530 |
| Potash | 8.286 |
| Soda | 10.908 |

| | |
|---|---|
| Chloride of Soda | 0.249 |
| Organic acid | 3.400 |
| | 97.000 |

This is a most elaborate analysis—far more minute than any analysis we have had of any of the articles of food—in fact, more minute than satisfactory; for the analysis of the whole kernel does not exhibit any amount of inorganic constituent; and when the whole was converted into ashes, we find that the *lime* only amounts to *the one-sixth of one part* in a hundred. Now, on inquiry, I find, on the authority of a very intelligent miller of this city, that in grinding corn, the bran, or thin skin of the grain, is detained in forming it into corn-meal; consequently, is deprived of even that portion more particularly containing the earthy constituents. This gentleman in conversation mentioned an important fact, relative to this deficiency of lime in corn. To the best of my recollection, he observed, "This stands to reason; for, ten years ago, all the lower part of Jersey grew excellent corn, but would not grow wheat; but since the introduction of *lime* as a manure, they have raised considerable wheat crops." Now the fact is, it is not the habit or food of this plant, even had *lime* been in the earth; and magnesia and the saline manures are recommended to the agriculturist as best suited for its proper development.

It is generally looked upon as invidious, and one is more likely to incur odium, than to receive credit for saying one word against a food which stands so high in public estimation, and is so universally used over this continent. Yet it must not, for one moment, be supposed that I condemn the use of Indian corn, in its various forms as mush, hominy, bread or pudding, as an article of diet: far from it. But containing, as it does, a large proportion of starch and fatty matter, rather a small proportion of the nutrient principle, and quite a deficiency of the inorganic or earthy constituents, I consider it as valuable, as a light diet, for heat-sustaining purposes only, and therefore a desirable adjunct *to other food*, containing more nutriment and a due proportion of the earthy constituents.

As an example or illustration of the want of the nutrient principle in corn or corn-meal, I may here allude to the effects I have seen in the West Indies; where, in a dearth of the ordinary provisions on which prisoners were fed, corn-meal was substituted; corn-meal and salted herrings, fish, etc., constituting their food. Now the effect was, that all the prisoners lost their natural strength; at the same time they became fat and bloated, inclining to dropsey; and this was not the effect of incarceration; for the prisoners were engaged in road-making, trimming fences, etc.; consequently, in a healthy and exhilarating employment.

In reference to cur domesticated animals, it may be asked, Why is corn so useful, as an article of food, to animals generally—horses, hogs, sheep, etc.? I have already shown that the overplus of the calorifacient food, after what may be required for sustaining the temperature, it stowed away in the form of fat. Now, if we instance the horse: corn is generally, if not always, given as an adjunct to his more usual food, hay. And we find by analysis, that grass or hay contains not only the nutrient principle, but the inorganic constituents required in the formation of bone, etc.

One hundred parts of dry hay contain—

| | |
|---|---|
| Carbon | 45.8 |
| Hydrogen | 5.0 |
| Oxygen | 38.7 |
| Nitrogen,* | 1.5 |
| Ashes,† | 9.0 |
| | 100. |

Thus, the hay gives to the animal strength in bone and muscle, while the corn supplies additional heat-sustaining properties, and lays by, in the form of fat, the overplus as a reserve. The harder the horse is worked, the more corn he can bear; the great proportion of the carbon being carried off by the lungs, and the hydrogen and oxygen, as water, in exhalation and perspiration. But if the same quantity is given to a horse at rest, it overloads him with fat, which, in his case, accumulates more internally, or around the internal organs, and will, in course of time, induce disease; while in the pig, under similar circumstances, the fat is laid on externally, if I may so speak, giving the rich fat pork of our markets. And here I would again remark, that no farmer would consider it necessary or essential to give corn to a young colt or horse, until required to work; nay, so careful is nature, in appropriating just so much and no more of any constituent that may be required, that the food of the young horse should be more nutritious than heat-sustaining; and that there shall be no superfluity to store away fat, we find by analysis, that the milk of the mare has little or no butter,

* Fifteen pounds of such hay, containing oz. 3.095 of nitrogen.
† These ashes having a good proportion of lime.

in fact only traces of it, in its composition.‡ What a lesson in the animal economy is here given, and what a practical illustration of the requirements of the young of that and other animals!

Again, it may be contended, that among the beautiful children we see on every hand, there is no want of those who are fat and hearty. It is not *fat* we want—it is bone and muscle—with so much fat only as shall give firmness to the flesh and plumpness to the figure. Fat, although it enters intimately into union with the other component parts of bone and muscle, cannot be transformed either into the inorganic constituents of bone or teeth, or into muscular fibre; these must be contained in the food consumed, in the first place, and thence transferred to the blood.

How necessary, then—how important is—if we expect to give strength and vigor to the constitution, that the food, in the first years of infancy and childhood, when the formative process is going on, should receive some further attention than has hitherto been given to it; and if our youth—if our young females have hitherto been deprived of the necessary constituents for the full development of every portion of the body—can we wonder that a woman should be the delicate and fragile being she is, or that by the decay which assails the teeth in early life, she should be deprived of an ornament of so much value? If this state of things can be altered—if the physical constitution of woman in America can be saved from further degeneracy—a purpose may be effected, of consequence even in a national point of view; for it is to the healthy and vigorous constitution of woman that we must look for a race of hardy, vigorous, and enterprising freemen.

In conclusion I would briefly state, that this is a matter in which professional aid can avail little; it lies at the door, and must be the work of parents generally. It is for them to understand the great value to be attached to the food on which their children subsist—that it shall be wholesome and nutritious, and abounding in earthy compounds so absolutely necessary to their proper development. If the chief articles of food have hitherto consisted of compounds made of superfine flour, corn-meal, and the fat of meat, let there be substituted in their stead, bran-

‡ ANALYSIS OF MARE'S MILK.

| | |
|---|---|
| Water | 896.3 |
| Butter | Traces. |
| Caseine | 16.2 |
| Sugar of Milk, Extractive Matters, and Fixed Salts | 87.5 |
| | 1000. |

bread, milk, eggs, the lean of meat, and potatoes; let more attention be given to the nutrient quality of the food;—let there be no deficiency of those articles containing the earthy material, that the bones and teeth shall not be deficient in those constituents so necessary in their composition and structure; and I should be inclined to hope that the evils which now exist will be lessened, and the physical organization of succeeding generations be equal to that of my nation upon earth.

